# Air Pollutants Removal Using Biofiltration Technique: A Challenge at the Frontiers of Sustainable Environment

**DOI:** 10.1021/acsengineeringau.2c00020

**Published:** 2022-06-03

**Authors:** Karamveer Sheoran, Samarjeet Singh Siwal, Deepanshi Kapoor, Nirankar Singh, Adesh K. Saini, Walaa Fahad Alsanie, Vijay Kumar Thakur

**Affiliations:** † Department of Chemistry, M. M. Engineering College, Maharishi Markandeshwar (Deemed to be University), Mullana-Ambala, Haryana 133207, India; ‡ Department of Biotechnology, Maharishi Markandeshwar (Deemed to be University), Mullana-Ambala, Haryana 133207, India; § Department of Clinical Laboratories Sciences, The Faculty of Applied Medical Sciences, Taif University, P.O. Box 11099, Taif 21944, Saudi Arabia; ∥ Biorefining and Advanced Materials Research Center, Scotland’s Rural College (SRUC), Kings Buildings, West Mains Road, Edinburgh EH9 3JG, United Kingdom; ⊥ School of Engineering, University of Petroleum & Energy Studies (UPES), Dehradun 248007, Uttarakhand, India; # Centre for Research & Development, Chandigarh University, Mohali 140413, Punjab, India

**Keywords:** Biofiltration techniques, Pollutants removal, Moisture content, VOC control, Residence time, Sustainable environment

## Abstract

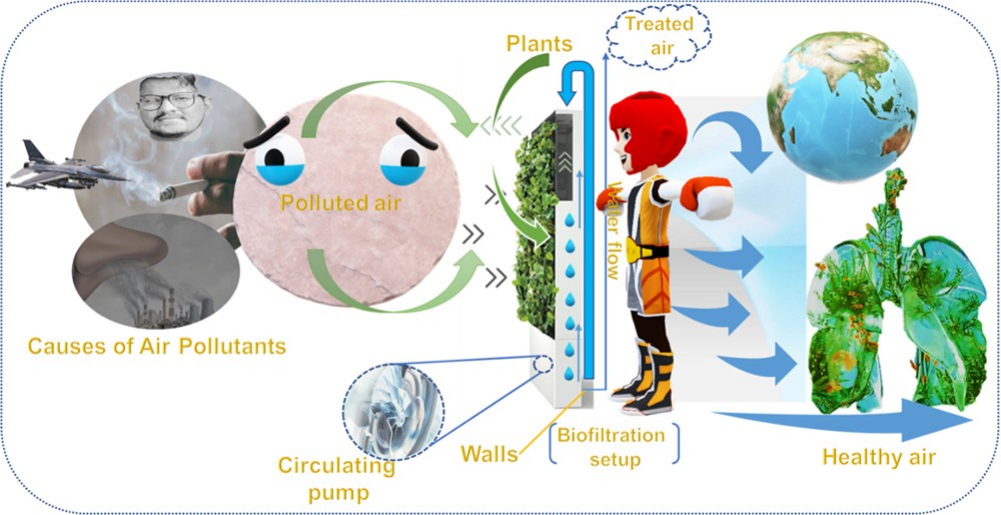

Air pollution is a central problem faced by industries during the production process. The control of this pollution is essential for the environment and living organisms as it creates harmful effects. Biofiltration is a current pollution management strategy that concerns removing odor, volatile organic compounds (VOCs), and other pollutants from the air. Recently, this approach has earned vogue globally due to its low-cost and straightforward technique, effortless function, high reduction efficacy, less energy necessity, and residual consequences not needing additional remedy. There is a critical requirement to consider sustainable machinery to decrease the pollutants arising within air and water sources. For managing these different kinds of pollutant reductions, biofiltration techniques have been utilized. The contaminants are adsorbed upon the medium exterior and are metabolized to benign outcomes through immobilized microbes. Biofiltration-based designs have appeared advantageous in terminating dangerous pollutants from wastewater or contaminated air in recent years. Biofiltration uses the possibilities of microbial approaches (bacteria and fungi) to lessen the broad range of compounds and VOCs. In this review, we have discussed a general introduction based on biofiltration and the classification of air pollutants based on different sources. The history of biofiltration and other mechanisms used in biofiltration techniques have been discussed. Further, the crucial factors of biofilters that affect the performance of biofiltration techniques have been discussed in detail. Finally, we concluded the topic with current challenges and future prospects.

## 1 Introduction

Air contamination is one of the severe issues of today, degrading the environment’s health. Many of the pollutants are carcinogenic, causing cancer and tumors, deteriorating human health and the environment. Many techniques are used to eliminate air pollutants like chemicals and microfilters, but they are costly and require maintenance.^1−3^ Biofiltration is the alternative technique, which can be used to remove air pollutants emitted mainly from organic product-based companies, for example, paint industries, pharmaceutical industries, and also by vehicles, municipal sources, substance adjustment landfill-related procedures, delivering plants, synthetic assembling processes, shops that print, flavors and scents, espresso and cocoa broiling, sewage treatment (smell evacuation), covering processes, fertilizing the soil, food handling, animals ranches, and foundries.^4−11^ Paint application and manufacturing companies utilize solvents which are the major, about 60%, pollutant generator. It is economical to remove pollutants and requires less maintenance.^12−15^

One of the main aspects is that bacteria effectively remove pollutants, but fungi can enhance degradation, mainly in paint application and manufacturing emissions. Fungi have a better removal efficiency for toluene used as a solvent in producing paints, gums, pitches, and elastic and utilized as reagents in developing medications, colors, and fragrances.^16^ Biofilter and biotrickling filters can be used as both are capable of removing hydrogen sulfide (H_2_S), odor, a wide range of VOCs^17^ (including chlorinated and nonchlorinated species, ketones, organic amines, aldehyde, ether, toluene, and aromatic hydrocarbons), and many other pollutants. However, VOC emission is comparatively less than H_2_S, a significant cause of malodor; ammonia is also responsible for malodor mainly produced from food processing and petrochemical refining industries.^18^ Moreover, it can remove carbon disulfide (CS_2_), which is generated when cellulose-based outcomes are produced (e.g., cellophane, rayon fibers, and cellulose sponges).^19^ It is efficient for readily degradable pollutants, for example, toluene, xylene, butanol (C_4_H_9_OH), formaldehyde (HCHO), trimethylamine, and acetaldehyde (CH_3_CHO).^20^ It also can remove volatile inorganic compounds (VICs).

Biofiltration is the alternative technique, which is a biological process requiring low maintenance cost, is more effective, generates lower amounts of harmful byproducts, and has a wide variety (range) of applications.^21^ Its performance can be affected by changing temperature, moisture content, and discontinuous pollutant supplies.^22−24^ The removal efficiencies for H_2_S degeneration are, for the most part, comparable to VOC contaminates; the convergences of specific VOC types are inferior.^25−27^

VOCs, like toluene, are industrial compounds grown broadly around the globe. The high attraction of enhancing the VOC reduction technique proficiency is connected to odor emissions and newly documented intense damaging human health consequences. Actually, at low concentrations, toluene is carcinogenic, induces injury to the liver and kidney, paralyzes the primary nervous system, and induces hereditary impairment. Toluene has been broadly investigated as a standard combination within biofiltration. Different researchers have concentrated upon toluene reduction through biofiltration at high burdens.^28−30^

**Figure 1:**
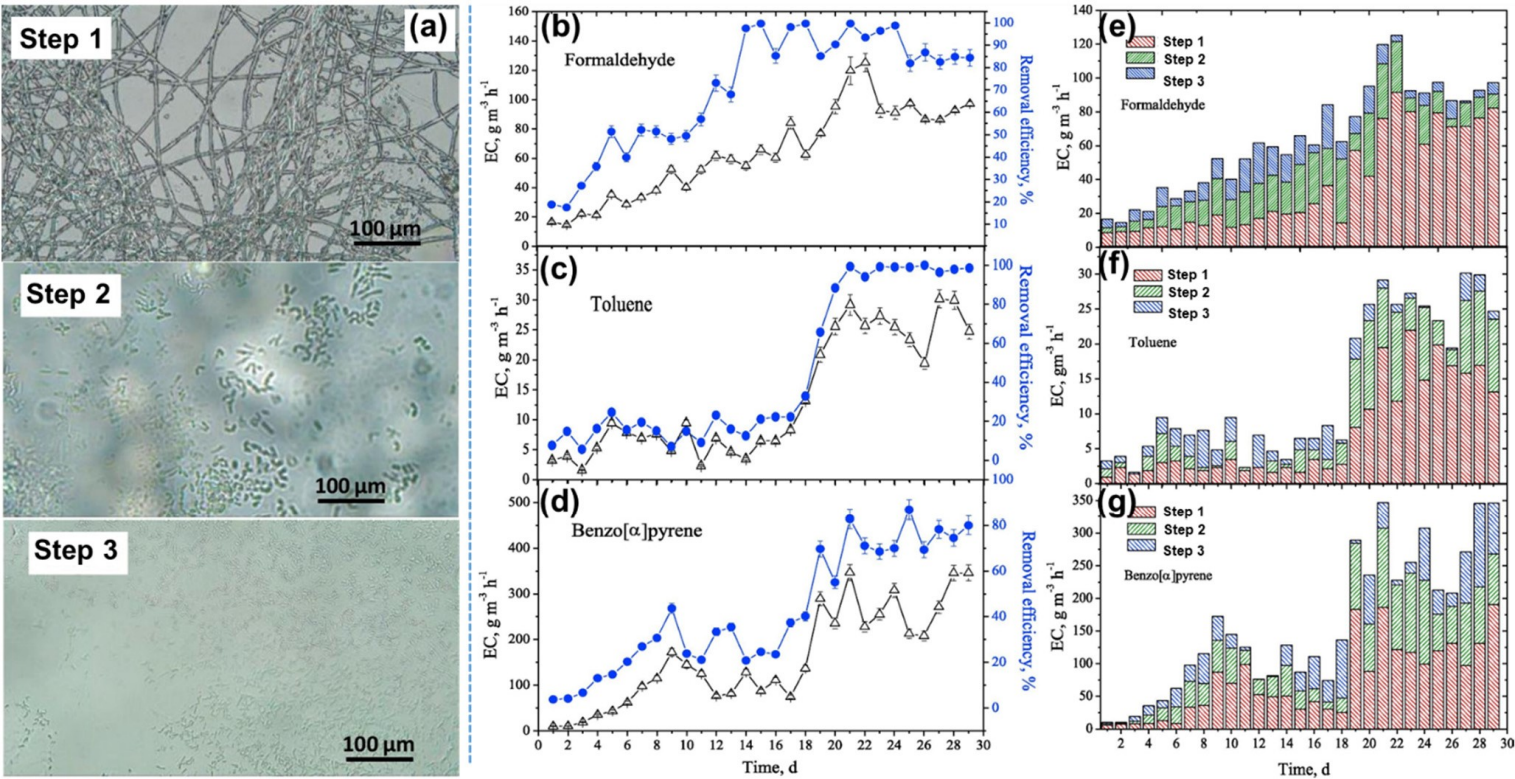
(a) Scanning electron microscope (SEM) pictures of specimens removed from the biofilter exhibit fungi and bacteria’ development within the various steps of the biofilter. Removal capability (empty triangle) and reduction efficiency (dark circle) in the start-up stage for formaldehyde (b), toluene (c), and benzo[α]pyrene (d) at 21 °C. (e–g) Development of the removal capability of individual impurities toward each step (1–3) during the start-up time. Reprinted with permission from ref (31). Copyright 2018, Elsevier Ltd.

In this regard, Vergara-Fernández et al.^31^ proposed that a study to maintain the moisture content (M/C) correctly was crucial to evade microbial deactivation. M/C was held beyond 60% with the acquisition of a mineral solution. Figure 1(a) demonstrates that step 1 was preferentially occupied with fungi, as was apparent in an explicit panorama with a dense fungal rug assembled. In the second and third steps, the fungal rug was missing. The removal capability at a constant state toward toluene achieved around 26.1 g m^–3^ h^–1^ (Figure 1(c)), 92.1 g m^–3^ h^–1^ toward formaldehyde (Figure 1(b)), and 320.8 g m^–3^ h^–1^ for benzo[α]pyrene (BaP) (Figure 1(d)). Elimination efficacy within the steady state was better, around 80% for formaldehyde, almost 100% for toluene, and nearly 80% for BaP. The stepwise removal capability was observed during the startup stage (Figure 1(e–g)) by estimating the medium concentrations of toluene, formaldehyde, and BaP into the step-departing outpour on every step.

In this review, we have discussed the general introduction based on biofiltration and the classification of air pollutants based on different sources. The histories of biofiltration and other mechanisms used in biofiltration techniques have been discussed. Further, the crucial factors of biofilters that affect the performance of biofiltration techniques have been discussed in detail. Finally, we concluded the topic with current challenges and future prospects.

## 2 Classification of Air Pollutants Based on Different Sources

Air pollution is one of the quickly rising issues of today’s world. Contaminants are ejected from various origins directly or indirectly to the environment. One or numerous contaminants also exist within the air for extended periods, which may have few detrimental effects on humans, cattle, and plants. This also influences the international economy and environmental transitions for long periods. Air pollution is currently viewed as the world’s most significant hazard to climate health and is responsible for seven million casualties worldwide every year. This generates several harmful consequences and induces pulmonary disease, asthma, and cardiovascular disorders after a long time period. Short-period times also cause headaches, mood change, dizziness, eye itching, sickness, coughing, and more.^32^ Air pollutants are categorized into the following different types.

### 2.1 Primary Air Pollutants

Pollutants acquired directly from their origin are primary pollution, for example, nitric oxides, sulfur oxides, particulate matter, carbon monoxides, and VOCs (see below).^33^ Many harmful air pollutants are transmitted from manufacturing plants, burning plants, public energy generation, commercial and residential combustions, and nonburning cycles.^34^ Natural sources include volcanoes, dust storms, and sea salt (which cannot be treated by biofilters or any other filtration, but these are in small amounts).

#### 2.1.1 Nitrogen Dioxide

Oxides of nitrogen are responsible for obtaining particulate matter. Nitric oxide (NO) is fashioned during elevated heat consumption of fuel (e.g., street vehicles, radiators, and cookers). Once these combinations go through the air, NO_2_ is produced. Stages are most noteworthy in metropolitan regions as it is a traffic-linked toxin.

#### 2.1.2 Sulfur Dioxide (SO_2_)

Fossil fuel ignition (generally energy places), change of wood pulp to paper, sulfuric acid (H_2_SO_4_) production, refining, burning of waste form sufur dioxide. The most well-known natural source is volcanoes.

#### 2.1.3 Carbon Monoxide (CO)

CO forms when carbon fuels are burned, either within the existence of too little oxygen or at very high heat.^35^ One of the fundamental causes is idling vehicle motors and vehicle deceleration. A lower amount is put into the air from natural burning in surplus incineration and energy station procedures. Levels are most noteworthy in metropolitan regions because of street traffic.

### 2.2 Secondary Air Pollutants

These pollutants are obtained by the reaction of primary pollutants and the atmosphere; examples include ozone and peroxyl acyl nitrates. Smog is a type of air contamination; “smog” is a combination of smoking and mist. A typical breakdown is produced from a lot of coal consumption in a space brought about by smoke and SO_2_. However, current smog does not generally come from coal but from vehicular and modern outflows that are put into the air and with daylight form secondary toxins that join with the essential emanations to form photochemical smog.^36^

#### 2.2.1 Ground-Level O_3_ Prepared from NOx and VOCs

**Figure 2:**
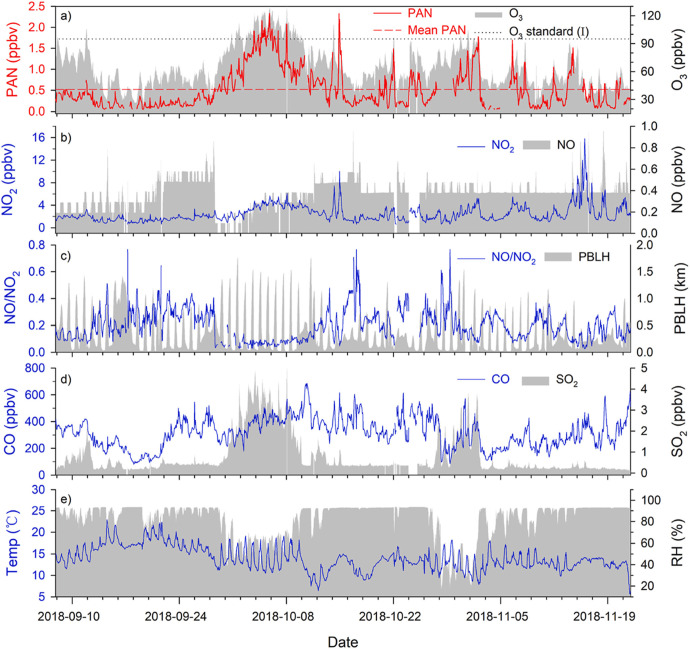
Time sequence of hourly PAN, O_3_, NO_2_, NO, NO/NO_2_, CO, SO_2_, heat, RH, and PBLH from 7 Sep–21 Nov 2018. Reprinted with permission from ref (38). Copyright 2021, Elsevier Ltd.

Photochemical and synthetic reactions initiate a large amount of the composite sequences, which occur within the environment by day and everywhere in the evening.^37^ At strangely high amount attained by humans (usually the ignition of petroleum), it is a toxin and a component of smoke. Peroxyacetyl nitrate (PAN) is also formed from NOx and VOCs. Figure 2 illustrates material interpretations of hourly PAN, trace fumes (O_3_, NO_2_, NO, CO, and SO_2_), the NO/NO_2_ proportion, and meteorological parameters (like heat, relative humidity (RH), and planetary boundary layer height (PBLH)) for the entire sample time on Mountain Tianjin (Mt. TJ).^38^

### 2.3 Toxic Organic Micropollutants

Polycyclic aromatic hydrocarbons (PAHs), polychlorinated biphenyls (PCBs), dioxins, and furans formed through the partial burning of fuels, street transportation, and modern manufacturing plants are the most significant cause of organic pollutants. Tobacco smoke is additionally a source.^39−41^ Urban air pollution is generally a consequence of burning petroleum products utilized in conveyance, energy production, industrial areas, and other financial actions.^42^

Household air pollution (HAP), also called indoor air pollution (IAP), is a critical area of concern in rural spaces, as a more significant part of this population relies on conventional biomass for cooking and space heating. Paraffin or additional liquid oils are also used for igniting, all of which can form primary to high stages of HAP.^43^ Over 70% of the residents of India rely upon old-style fuels (wood, crop deposits, cow dung, and coal) to cook their food, and nearly 32% depend upon kerosene for illumination purposes. Around 3 billion people (over 40% of the worldwide population) rely on traditional biomass to cook, and an expected 500 million families depend on paraffin, which is comparable to igniting. In the countryside of India, for example, just 11.4% of the families use LPG for cooking.

Parameters of air quality from the World Health Organization (WHO) focus on four health-correlated air pollutants, PM, estimated as particles with an aerodynamic width lower than 10 μm (PM_10_) and lower than 2.5 μm (PM_2.5_), NO_2_, SO_2_, and O_3_. The emphasis on these four is for observing the overall condition of air quality, and it does not imply that the other air poisons do not affect the health of people and that of the climate.^44^ Benzene, 1,3-butadiene, HCHO, vinyl chloride, perchloroethylene, and PAHs are cancer-causing air poisons. Benzene might be the most remarkable natural cancer-causing agent because the International Agency for Research on Cancer has characterized it as the Group 1 cancer-causing agent (affirmed as a human cancer-causing agent).^45^

Relevant measures in Japan taken to reduce HAPs include taking essential steps to decide the situation with outflow and release of HAPs into the air:^46^

- Studies will be carried out with local public substances to decide the situation with air contamination through HAPs. It shall occasionally give the community the human health hazard assessment results.
- The Air Pollution Control Act was passed to control soot emission, smoke, particulates, VOCs, perilous air contaminations, and engine vehicle exhausts.
- On the basis of the cancer-causing nature, physicochemical properties, and checking of information, benzene, trichloroethylene, tetrachloroethylene, and dichloromethane were first assigned as HAPs.

The Environmental and Financial Ministry, Trade, Industry in Japan set up a “Guideline for promoting Voluntary Control of Hazardous Air” to control the assigned substances, including benzene and trichloroethylene contaminants, through commercial units.” Under this rule, every manufacturing group from one side of the country to the other created a voluntary reduction plan in 2003. The Ministry of Environment (MOE) has ordered the results of the monitoring survey to be made public. The fixation levels of four poisonous VOCs fundamentally showed a diminishing pattern during this time.

The central administration also shall establish measuring systems and continuously calculate the class of air contamination:^47^

- According to the installation control standards, acceptable emission levels, lowering facility structure and function, leakage monitoring, and keeping standards will apply to every enterprise.
- To diminish the health hazard of cancer-causing VOCs from their ephemeral emission, counteraction, and controller, the executives’ guidelines for HAP-producing offices authorized under the Clean Air Conservation Act’s correction have been successfully started on 1 January 2015. The board norms incorporate reasonable outflow levels, lessening the abilities of establishment and operation, and leak control and preservation standards in this office.

### 2.4 Main Sources of Air Pollution

According to the National Ambient Air Quality Standards (NAAQS), air pollutants such as PM_2.5_, SO_2_, NOx, CO, and O_3_ are usually higher in the atmosphere. With industrial emissions, vehicles and fuels in domestic use also contribute to the generation of pollutants, as most households contain two-wheel and four-wheel vehicles. There are still many homes using traditional power that cause health hazards, such as kerosene, biomass, and coal, that contributing to pollutant emissions, although many switched to liquefied petroleum gas (LPG). With the generation of electricity and its use and alternate power generation sources such as in situ generation (i.e., coal, diesel), the industries load of pollution generation will increase. An increase in air pollutants leads to an upsurge mainly in cases of diseases like ischemic heart illness (that may be the reason for heart attacks), cerebrovascular diseases, chronic disruptive lung disease, lower breathing contaminations, and cancers (trachea, lungs, and bronchitis).^48,49^

## 3 History of Biofiltration

Microbial reactions in soils usually happen for a long time; however, since the 1950s, such strategies have been utilized to treat waste gases.^50^ The biofilter was first discovered by German scientist Bach in 1923. Over time, biofilters and bioreactors have been adopted as typical ways of controlling pollution. Richard Pomeroy received U.S. patents in 1957 for a Long Beach soil bed concept. He described a practical soil bed set up in California.^51^ The first successful files and copyrights of biofilters were conveyed in the initial 1950s together the United States and Germany.^52^

The predominance of patent action did not begin until the late 1980s and initial 1990s, although there was proof of the overall inactivity in the biofiltration arena for the numerous years subsequent Pomeroy’s discovery.^53^ Carlson and Leiser showed the original orderly investigation of biofiltration of H_2_S in the mid-1960s. Their study reported the effective establishment of a few soil filters at a wastewater processing plant close to Seattle. It confirmed that biodegradation is slightly more than sorption described for the odor elimination. A large part of the information about the innovation is due to Hinrich Bohn, who has examined soil bed theory and had for over 15 years successful soil bed applications in the U.S. that incorporated the control of odors from rendering plants and the destruction of propane and butane from an aerosol filling operation.^54^ Before adapting this to agriculture, biofilters were utilized in wastewater treatment plants, chemical assembling facilities, soil fertilization, and other industrial air pollution schemes. They were first valuable for livestock facilities in Germany in the 1960s to reduce order emissions.^55^

During the 1960s and 1970s, biofilters were effectively utilized within West Germany to resist smells from various causes, such as sewage processing plants, fertilizing soil, food treatment, and chicken and pig ranches. Different plans were examined for the air circulation framework and a few sieve constituents with higher natural exercises and lower flow resistance than soil. Fertilizer from municipal solid waste (MSW) was utilized as a sieve substance in 1966. It was also recognized is a requirement for humidification of the off-gas at developed stream rates. The essential cycles defining the effectiveness of a filter were seen during the 1960s. Since the mid-1980s, Germany has progressively utilized biofiltration to control VOC and air pollutants radiated from manufacturing plants, for example, biochemical plants, factories, print workshops, and covering processes. It controls odor from wastewater treatment plants, animal rendering plants, and solid waste treatment. After a long research period, the biofilter is now used to treat from a simple single compound containing gas (methanol) to a mixture of contaminants (BTEX).

Currently, the processing of VOCs from soil cleaning activities has been tended to in a few studies. It very well may be derived from the absence of studies available within the U.S. Throughout the most recent 20 years, little consideration has been paid to simultaneous growths in two European nations: Germany and Netherlands. Within these nations, biofiltration has been used since the mid-1960s and developed into a broadly utilized APC innovation which is currently viewed as the best accessible controller technology (BACT) in an assortment of VOC and scent monitor applications.^56^ Thus, when developed and used correctly, biological methods present advantages including cost effectiveness, reliability, strong performances, and eco-friendliness over traditional approaches, for example, physicochemical adsorption, condensation, incineration, and photolysis. Lately, biological methods have become increasingly appealing and competitive, in which bioscrubbers, traditional biofilters, biotrickling filters, and unique biofilters have been employed or formed.

### 3.1 Important Points about Biofilters

The packing material should be chosen carefully because it affects the biofilter’s overall cost and size. Its particle size should be according to contaminants. (Prior to the general dimensions of the biofilter being determined, it is helpful to recognize an appropriate solid bed material since the material of choice will affect the overall working cost of the filter, just as the required size).^57^

This could improve the general activity of the filter bed by adding inactive solids like polystyrene beads to decrease compaction, broaden bed life, and increase absorbency.

#### 3.1.1 Health and Safety Concerns

There have been few investigations on the probable well being and care with the use of biofilters. The dependence on natural microbes in manure, soil, or fertilizer will cause people sensitive to these organisms to wear a facial covering to limit contact with airborne bacteria and mold microorganisms. Breathing assurance is suggested during development, upkeep, and media elimination.

### 3.2 Biofilter Setup

Biofilters consist of a humidifier or humidification chamber, a packing media reactor, and a particulate collector that collects particulates before gas is vented through a biobed (approximately 1 m deep) to distribute gas uniformly.

**Figure 3:**
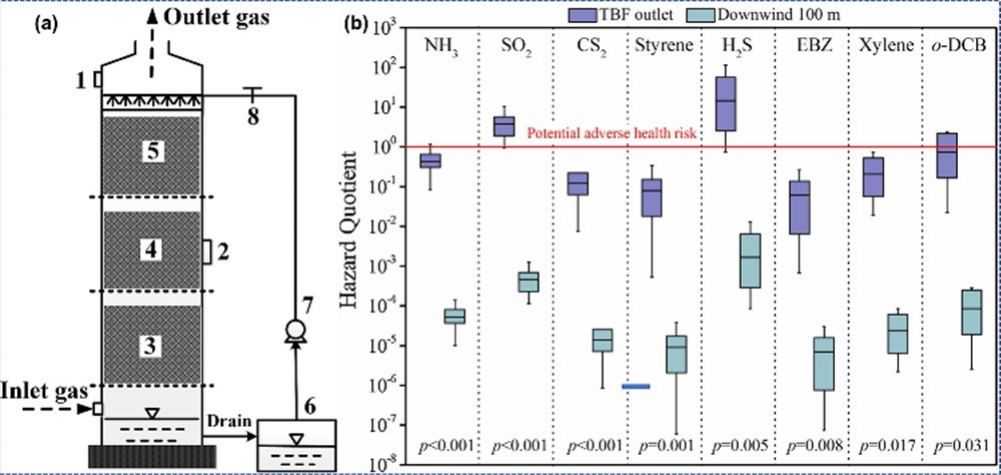
(a) Graphic illustration of the TBF: (1) gas and bioaerosols specimen collections, (2) stuffing substance sample ports, (3–5) PUFCs, (6) nutrient container, (7) pump, (8) regulator. (b) Health threat from exposure to NH_3_, SO_2_, and six main VOCs in the TBF opening and 100 m leeward. CS_2_, carbon disulfide; EBZ, ethylbenzene. Reprinted with permission from ref (58). Copyright 2019, Elsevier Ltd.

Yang et al.^58^ studied the impact aspects and health threats of inspection of bioaerosols radiating from an industrial-range thermophilic biofilter (TBF) toward off-gas therapy. The TBF-treated sludge aeration fan contains SO_2_, NH_3_, and complete VOCs. It included a stainless-steel support with a height of 25 m and an inner diameter of 2.0 m (Figure 3(a)). At 100 m leeward, the median threats of SO_2_, H_2_S, and 1,2-dichlorobenzene (*o*-DCB) were 4.61 × 10^–4^, 1.67 × 10^–3^, and 7.01 × 10^–5^, respectively, and the extreme dangers were 1.22 × 10^–3^, 1 × 10^–2^, and 4.34 × 10^–4^, respectively (Figure 3(b)).

Different methods have been designed to reduce methane (CH_4_) emissions, as CH_4_ is a potent greenhouse gas. Biological filtration is utilized for CH_4_ alleviation from dumps, coal mines, and animal farming where CH_4_ is ejected. Aerobic CH_4_-oxidizing bacteria (methanotrophs) employ CH_4_ as their exclusive carbon and energy origin^59^ and reduce CH_4_ during CH_4_ percolation. Earlier investigations of CH_4_ biofiltration have primarily concentrated on abiotic aspects, for example, bed substances, heat, loading rate, and pH.^60−62^ Several materials, such as perlite, granulated activated carbon, and compost, have been considered filter beds for CH_4_ reduction.^63^ Lately, biological factors, such as microbes, have increased awareness in CH_4_ biofiltration analyses.^64^

## 4 Biofiltration Technique

A biofilter for controlling air toxins comprises at least one bed of biologically active material; essentially, a mixture dependent upon manure, fertilizer, or soil filter beds is commonly 1 m in height. The polluted off-gas is vented from the producing source through the filter. In a specific adequate time, the air pollutants will diffuse within a wet, biologically active layer (biofilm) surrounding the filter particles. Aerobic degradation (AD) of the target will happen in the biofilm if microbes, fundamentally microorganisms, are available that may use them. The total biodegradation of air pollutants is CO_2_, water, and bacterial biomass.^65,66^ The oxidation of decreased sulfur complexes and chlorinated organic mixtures creating inorganic acid compost, for the most part, made from city surplus, wood pieces, bay, or leaves has commonly been the premise of sieve substances utilized in current applications in Europe, even though compost and a heather mixture have additionally been used. Initially, the biofilters in the built in the U.S. were generally “soil beds” for which biologically active mineral soils were utilized as sieve constituents.

**Figure 4:**
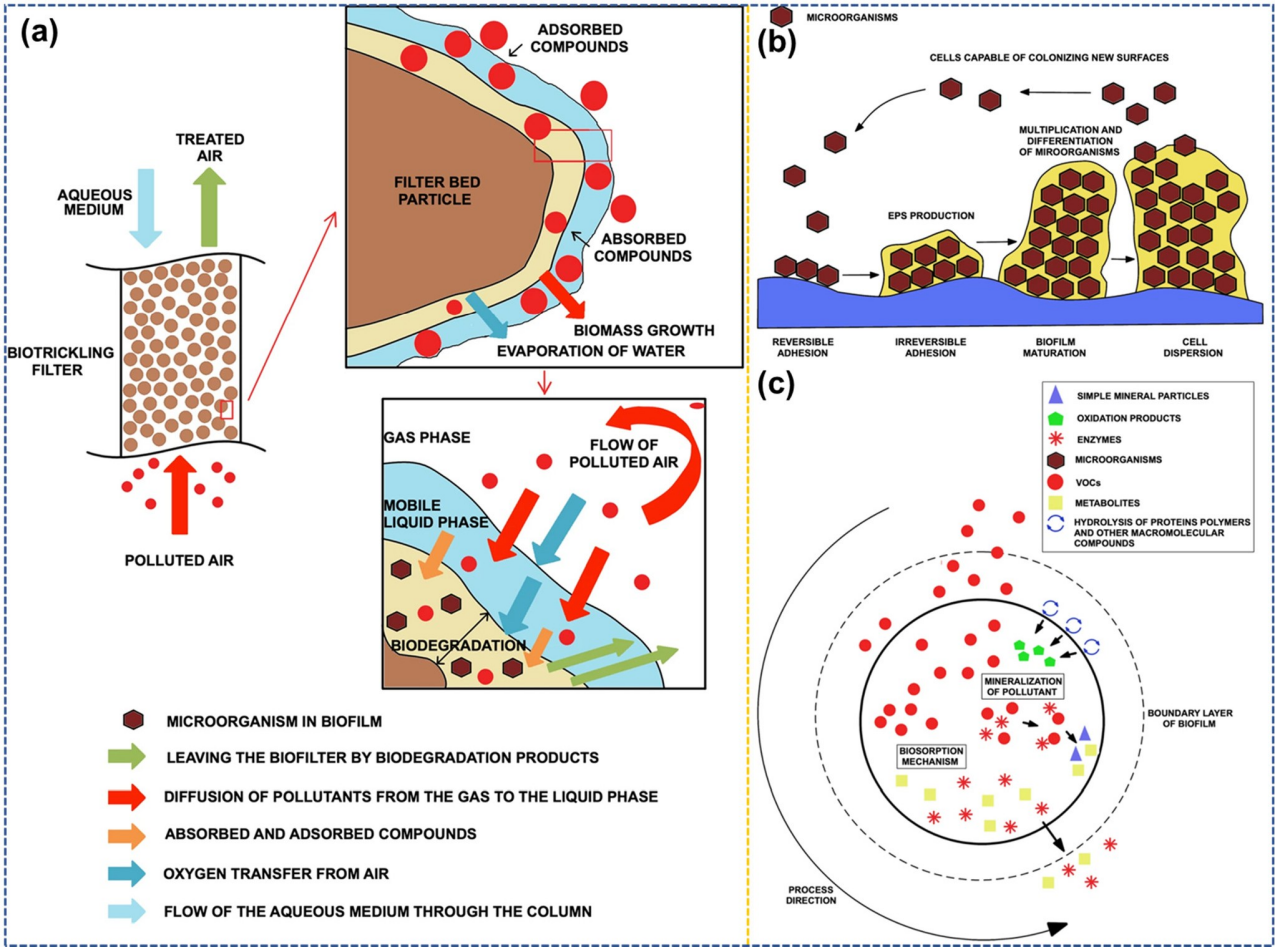
(a) Available tool of gas contaminant reduction in biotrickling filtration. (b) Steps of biofilm appearance in biofiltration systems. (c) Physiochemical tools within biosorption and mineralization of contaminants. Reprinted with permission under a Creative Commons CC BY License from.^67^ Copyright 2022, Springer Nature.

Marycz et al.^67^ proposed a biofiltration study on fungi to dismiss volatile hydrophobic contaminants. The removal of gas impurities in biofiltration results from an intricate blend of different biological and physicochemical spectacles (Figure 4(a)). The procedure of air sanctification through biological techniques applies microbes, most often bacteria and fungi, to deteriorate the VOC into nontoxic constituents. Figure 4(b) shows the four significant steps of biofilm construction. Suspended fungal cells adhere to the column’s bed filler surface within the first step. The foremost one, named biosorption, entraps the gas contaminants on the exteriors of microbe cells. A bidirectional interaction ensues: contaminant molecules diffuse within the cells, although enzymes and metabolites transit into the contrasting path (Figure 4(c)).

### 4.1 Use of Biotrickling Filters

Biotrickling filters are better than average (conventional) biofilters because of their continuous changing of eluent (fluid rivulet of water with or without extra supplements practical to the intense media), resulting in reseeding of microbes, controlled pH, and therefore increased efficiency of the biofilter. A continuous water supply reduces the acidification of the bed, which results from the acidic byproduct of degradation of CS_2._^68^ Elimination of CS_2_ is very low upon treatment with biotrickling channels introduced in rayon fiber and cellulose wipes.^69^

### 4.2 Use of Biofiltration Technique over Other Methods

Adsorption, thermal oxidation, catalytic oxidation, and chemical scrubbing are some of the techniques which are used in industries for the degradation of pollutants,^70,71^ but they have some disadvantages for dilute industrial VOC emissions:

(i) Adsorption technique: Activated carbon is used to adsorb VOC. Consequently, VOCs accumulate on activated carbon and thus form a new waste.
(ii) Thermal oxidation technique: In most industrial pollutant emissions, VOC concentration is comparatively less than other pollutants. Therefore, self-incarceration is impossible due to this external fuel being supplied for increased heat for degradation, making this technique expensive.
(iii) Catalytic oxidation technique: Catalytic oxidation can be clogged due to catalytic poisoning by the presence of chlorinated organic and sulfides.

### 4.3 Disadvantages of Other Techniques

Traditional treatment frameworks have high speculation costs, utilize significant energy measures, and produce waste streams (e.g., activated carbon or SO_2_ discharge). Other air contamination control innovations like adsorption and burning may be compelling in processing the VOCs. They can create undesirable side effects and may not be appropriate for taking care of a high flow toxin rivulet with a low concentration of pollutants.

### 4.4 Other Techniques for Removals of Pollutants

#### 4.4.1 Membrane Separation

**Figure 5:**
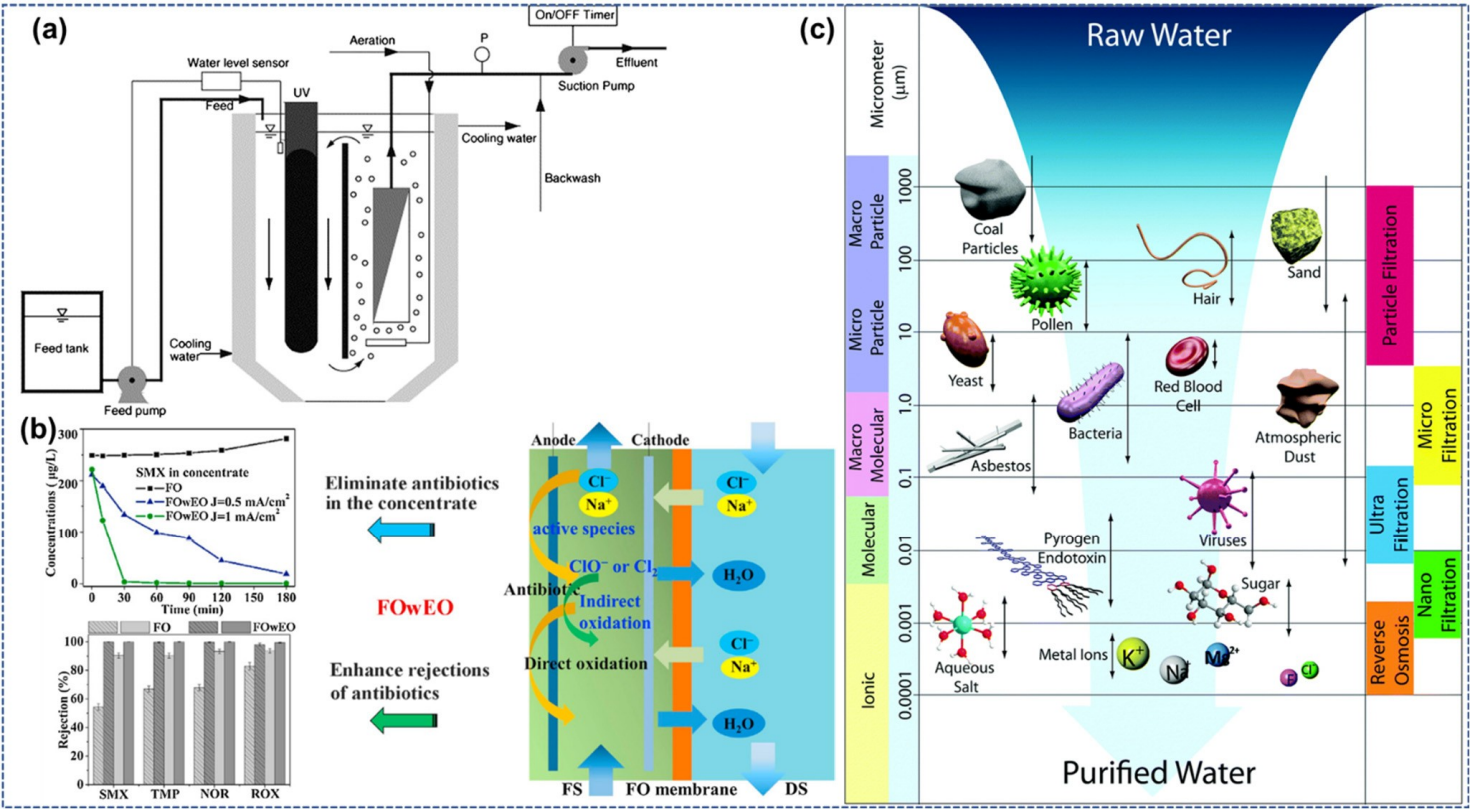
(a) Aquatic membrane photocatalytic device. (b) Graphics of Forward osmosis with electrochemical oxidation system (FOwEO) approach leading to improved denial and removal of antibiotics concurrently. (c) MBSP spectra, like method title, size range, and possible solute abandoned over the specified capacity of pores. Reprinted with permission from ref (79). Copyright 2019, Elsevier Ltd.

A membrane is a delicate material boundary that reconciles specific species to depart, relying upon their physical and/or chemical effects.^72,73^ Membrane-based separation procedures (MBSPs) are well-known detachment technologies that provide different applications in water desalination, poisonous metal cleavage, and retrieval of valuables.^74−76^ The membrane methods rely upon the essence of membranes made from various substances, like polymers and ceramics, zeolites, containing explicit filtering qualities, which depend on the exterior charge, pore size, and membrane surface structure hydrophobicity/hydrophilicity features.^77,78^ Studies have been completed on both systems of photocatalytic membrane reactors (PMRs), relying upon membrane modules. The immersed membrane photoreactors have been successfully employed to get clean water, as shown in Figure 5(a). A synergistic impact was followed within this hybrid approach where antibiotic denials with forward osmosis (FO) were raised owing to the removal of antibiotics when electrochemical oxidation (ECO) was enhanced through this process (Figure 5(b)). MBSPs are modules like MF, UF, NF, RO, and FO that use various membranes, relying upon their pore sizes, surface structures, and precise separation necessities, as shown in Figure 5(c).^79^

#### 4.4.2 Plasma Destruction

VOCs are pollutants from various origins, such as semiconductor engineering factories and chemical processing manufacturers. Their existence in the air adds to photochemical pollution creation; VOCs also contaminate the earth, drinking water, and groundwater. The ejection of VOCs into the ambient air is harmful to both humans and the atmosphere.

This hybrid plasma-catalysis approach, incorporating plasma and catalysis processes, has been broadly studied and grown recently.^80,81^ It is currently well proved that the execution of nonthermal plasma techniques to remove low concentrations of contaminants may be enhanced, mainly by counting catalyst substances in the combustion area of the apparatus. The performance of a plasma-catalytic instrument is incomparable to a plasma container toward a capacity of VOCs. The benefits of utilizing plasma-catalysis techniques over plasma alone include the improved transformation of contaminants, lower power intake, enhanced energy efficiency toward the plasma procedure, more elevated CO_2_ discrimination, and a prolonged catalyst lifetime.^82,83^ A synergistic outcome has been noted within a few matters for the plasma-catalytic deterioration of VOCs. In contrast, the joint processing consequence is higher than the sum of the respective phases. The enthusiastic species constructed through the nonthermal plasma have a high catalytic capability; their attention improves with growing plasma energy, indicating that the synergic outcome also increases with energy.^84^

#### 4.4.3 Ozone Catalytic Oxidation

Indoor air quality (IAQ) is a subject of significant general consideration because the lifestyle of individuals has transformed from open air to indoor recently; generally, people in urban regions spend around 80% of their duration within indoor circumstances. Therefore, governments have precisely controlled IAQ to safeguard human health. Indoor air contaminants are composed of various materials, such as VOCs, carbonyl complexes (CO, CO_2_), and bioaerosols. They are ejected from different origins like scorching and cooking, building substances, atmospheric surroundings.

Contaminants like sulfur oxides (SOx), nitrogen oxides (NOx), and other impurities are formed. At the same time, coal-fired energy production may induce moisture and acid rain. Various issues have powerful environmental influences like photochemical decay and ozone (O_3_). Consequently, individuals utilize different technological standards to facilitate many coal-fired emissions.^85^ As a gas oxidant, the typical redox voltage of O_3_ is 2.07 V, representing a solid oxidation execution and a prolonged survival period below low- and medium-heat circumstances (<270 °C) and delivers nontoxic O after deterioration. Large-range generation of O_3_ would be recognized via a dielectric barrier release reaction apparatus. These benefits create O_3_ oxidation technology sufficiently valuable for manufacturing wastewater remedies.^86,87^ In the domain of chimney gas multicontaminant synergistic reduction, O_3_ oxidation has also evolved as one of the technologies with usage options.

Catalysis is a very efficient technique (used for product formation to reduce emissions). Catalysis is utilized to stop contaminations from fixed origins like power factories, portable sources like vehicles, and progressively common conditions like offices, homes, and retail outlets.

## 5 Different Mechanisms Used in the Biofiltration Technique

There are two kinds of biodegradation frameworks (not biofilter). Microorganisms are delimited in a rinse fluid communicated with the polluted air and absorber within bioscrubbers. This part will emphasize biofilters, frameworks where the microbes are delimited on a solid substance, like fertilizer, soil, granular activated carbon (GAC), diatomaceous earth, or inactive synthesized substances. With flue gas, the pretreatment equipment biofiltration system varies by the number of beds, packing media used, and how the gas will distribute in the whole packing bed.^88,89^

### 5.1 Biofiltration of VOCs by Using Fungi

Environmental contamination has evolved into one of the main reasons for early demise within advanced and developing nations.^90−92^ While some other pollutants are sufficiently apprehended, like O_3_ generating an extra 0.25 million casualties, the effect of VOCs has not been thoroughly calculated, except with O_3_ appearance, which is usually related to PM and PAHs.^93^ VOCs contain organic compounds with an increased vapor pressure at ambient conditions and generally exist within indoor and outdoor atmospheres.^94^

**Figure 6:**
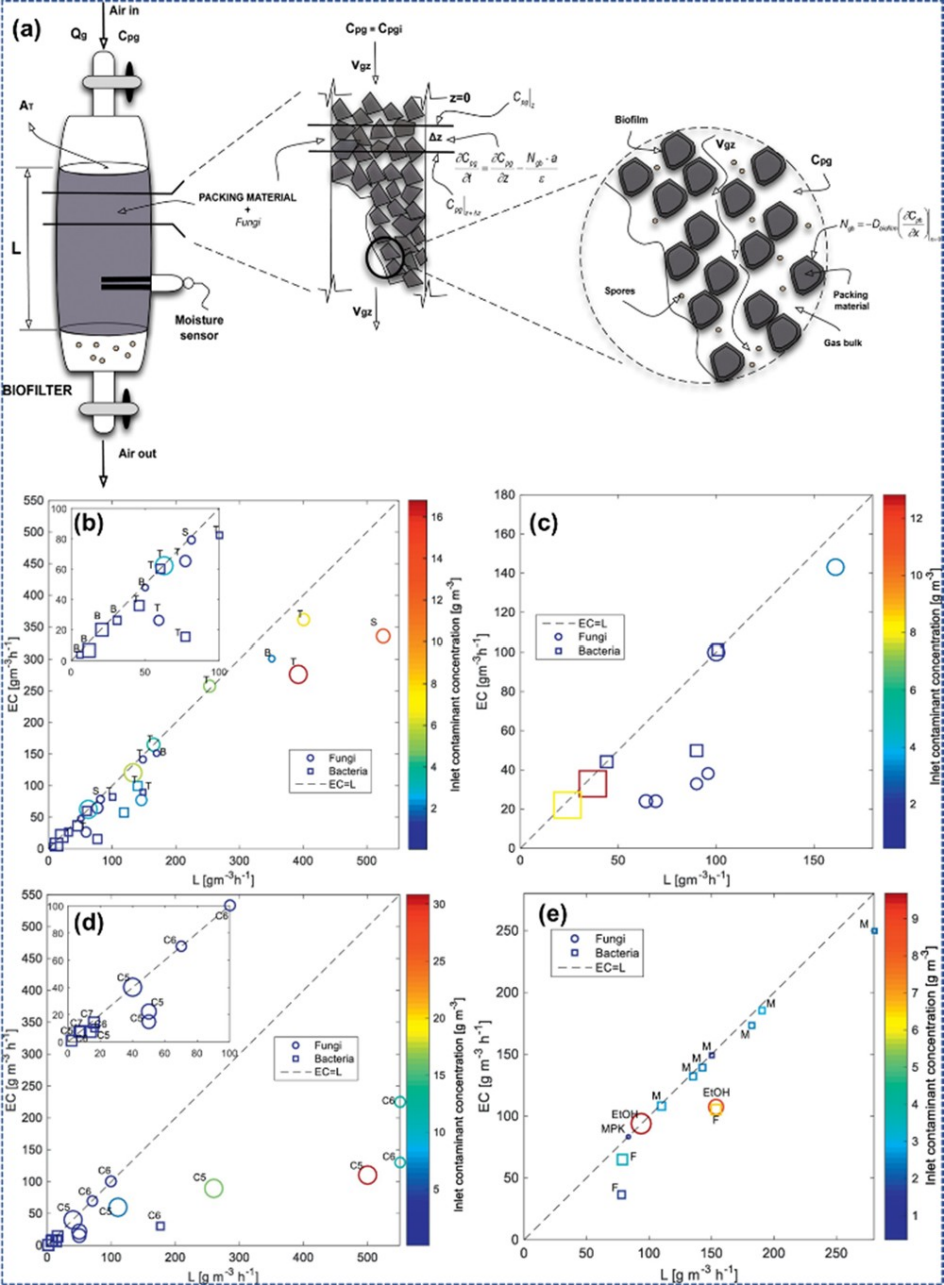
(a) Strategy of a conceptual sample of a fungus biofilter demonstrating the various hierarchies applied. Removal capability and load toward fungal (circle) and microbial (square signs) biofilters. (b) Biofilters treat benzene (B), toluene (T), styrene (S), and xylene (X). (c) Biofilters processing α-pinene. (d) Biofilters processing *n*-pentane (C5), *n*-hexane (C6), and *n*-heptane (C7). (e) Biofilters processing methanol (M), ethanol (EtOH), formaldehyde (F), and methyl-propyl-ketone (MPK). Reprinted with permission from ref (95). Copyright 2018, Elsevier Ltd.

In this regard, Vergara-Fernández et al.^95^ proposed a study based on the biofiltration of VOCs utilizing fungi and its theoretical and mathematical modeling. Figure 6(a) illustrates a notional standard of a biofilter. Pollutants are trapped by the air’s biofilter at paces that explain the laminar flow. These significances were utilized as shown in Figure 6(b–e), whereas the fungal biofilters may be noticed outperforming their bacterial replication within treating hydrophobic VOCs. In contrast, the information is lacking upon using fungal biofilters to abate hydrophilic combinations, and the available data reveal no distinctive benefits toward the fungal-established biofilters over microbial ones.

The use of fungi has an advantage over other microbes as they can work under low pH and changing moisture content.^96^ Fungi have been generally divided into six ordered divisions: *Zygomycota*, *Ascomycota*, *Basidiomycota*, *Chytridiomycota*, *Oomycote*, and *Myxomycetes*. Most fungi found in biofilters are *Ascomycota* and *Basidiomycota*. Fungi are heterotrophic and feed from nutrients in their environment; fungi secrete digestive enzymes to break down substrate and absorb nutrients. With ample surface area, fungi work better than volume.^97,98^ Fungi live in moderate temperature conditions, within pH ranges of 4–7, and a minimum of 70% water is required for fungal growth. Some fungi, such as species of Mucor, are drought tolerant. Fungi can live in less water than bacteria. Moreover, they can comparatively treat more VOC emissions, and the emission rate is equal to or greater than bacteria.

Fungi are suitable for treating a single component or a mixture of two components. Still, it is not confirmed whether they are well suited for a mix of an element or not, and paint manufacturing suggests that it may be better for treating solvent emissions.

### 5.2 Treatment of CS_2_ by *Thiobacillus thioparus* (Bacteria)

CS_2_ is a combustible organosulfur combination utilized continually as a building block within organic chemistry and a manufacturing nonpolar solvent. Considerable parts of CS_2_ are ejected into the environment while manufacturing cellulose-based outcomes (cellophane, rayon fibers, and cellulose leeches).^99^ These release parameters have been revised in the U.S. and Europe based upon their poisonous atmospheric effect and detonation risk. Presently, the methods to withdraw CS_2_ from contaminated vapors are standardly established upon captivation, adsorption, and thermal or catalytic oxidation.^100^ These traditional restorative methods have heightened asset prices, used significant energy, and generated trash streams. Recently, biotechnological trash processing techniques have progressively been utilized for industrial implementations because numerous disadvantages of classical physical–chemical processes may be overwhelming.

One of the significant expected functional issues within traditional biofilter processing of CS_2_ toxic vapors streams is the quiet start-up stage of the procedure. It is generated together through the microbial poisonousness of CS_2_ and because the biodiversity of microbes competent in metabolizing CS_2_ occurs to be highly narrow.^101^*Thiobacillus thioparus* is the only species of fungi that can degrade CS_2_ by growing on it and degrading CS_2_ to CO_2_ and H_2_S. Autotrophic metabolism of CS_2_ is connected to relatively low evolution rates by repetition times from 30 to 40 h in liquid batch cultures and could be used in sluggish bioreactor start-ups.

## 6 Important Factors of Biofilters That Affect the Performance of Biofiltration

Some vital parameters that impact the workings of a biofilter and microbial growth are moisture content, contaminants, nutrient concentration, loading rate, pH level, temperature, oxygen concentration, residence time, concentration of pollutants, and degree of contact between pollutants and biofilters.^102,103^

**Figure 7:**
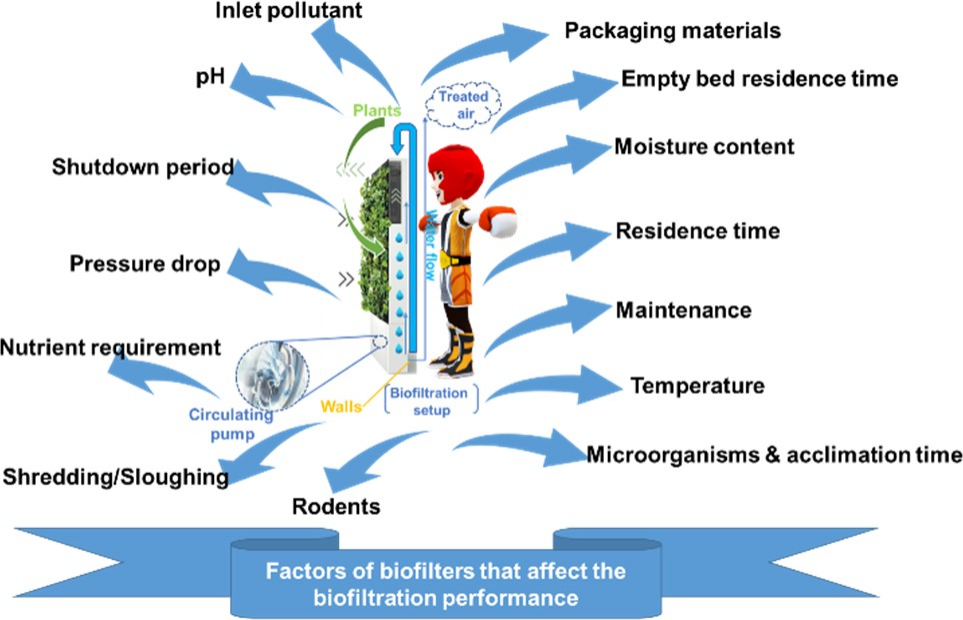
Essential factors of biofilters that affect the performance of biofiltration setup.

Biofiltration mainly depends on how many microorganisms are present in the biofilter. Microbes degrade contaminants either as primary metabolites or cometabolites. The boundaries that are utilized for communicating the presence of the biofilters are population loading capacity (L), elimination capacity (EC), and removal efficiency (RE). Figure 7 shows the crucial factors that affect biofiltration performance.

### 6.1 Packing Material

The central part of the biofilter is the bed of organic material containing compost, peat, or a similar soil, GAC or dirt, or inactive synthesized packing substances, which comprise perlite, pelletized ceramics, ceramics stones, diatomaceous earth, and stuffing media on which microorganisms attach.^104,105^ Contaminated gas or waste gas is first humidified and then passed through this packed media by manifold pipes to distribute gas uniformly. Contaminated gas may get adsorbed on biofilm where microorganisms degrade pollutants into harmless products, i.e., CO_2_, water, and cell mass. The central part of the biofilter is the packing media as it holds the biofilms, i.e., microorganisms.^106,107^

The media should deliver even air dispersal and pressure reduction via the bed, increased specific exterior area, better porosity, acceptable inorganic nutrients, adequate drainage, suitable mechanical power to rebel decay, negligible pressure reduction, and an exterior extension of the microorganisms. Aromatic compounds, such as benzene, could be removed from air streams in biofilters with animal waste compost as the filter medium.^108^ Media assortment is crucial in a biofilter enterprise. The media should give an appropriate climate for microbial development and keep a good absorbency to permit air to flow without any problem. Basic properties of media substances comprise (1) sponginess, (2) moisture-holding limit, (3) nutrient content, and (4) slow decay.

Biofilter media need to have from 50% to 80% voids to permit air to flow through without any problem. Numerous biofilters utilized within animal agriculture use a media which combines wood pieces and manure. Wood pieces offer mechanical help and void space. Waste gives a nutrient-enrich climate and is a primary cause of aerobic microbes.^109,110^ The latest investigation has confirmed that media composed basically of wood pieces covered in compost slurry or another microbe source are active and require less regular replacement. Other conceivable filter media incorporate wood bark, coconut fiber, peat, granular-initiated carbon, perlite, pumice, and polystyrene beads.

### 6.2 Moisture Content

Moisture content (M/C) should be adequate, i.e., not too low, which can result in drying of the bed with cracks appearing that can hamper the efficiency of microorganisms. Hence, untreated gas will escape through the bed, and dryness can also result from the process of biodegradation as it is an exothermic process and also by heat exchange by surroundings. Moreover, it should not be too high, which leads to water channelling and anaerobic conditions resulting in odor from the bed. M/C is controlled by humidifying the incoming air by 90%–95%. M/C can be examined by measuring electrical conductivity or capacitance in given spots, but mainly, “load cells” are used. However, we cannot use these in open biofilters due to the additional weight of vegetation growth, snow, and other factors. To maintain M/C, the gas flow should be downflow as the entrance surface is drier. Still, in the case of cyanide- and sulfide-containing products, it should upflow as the degraded acidic product can easily wash off from the bottom. The ideal M/C is, for the most part, viewed as around 35%–60% in fertilizer biofilters for eliminating H_2_S and VOCs.^111,112^ The fundamental driver of drying biofilter pressing materials is the fragmented humidification of the bay air stream and the metabolic hotness produced by poison bio-oxidation.^113,114^

### 6.3 Effect of Residence Time

As the biological process is slow and takes time for diffusion of gas, removal efficiency increases as the empty bed contact time (EBCT) increases. While bed channelling happens, the helpful connection among the biofilm is restricted, and the actual pollutant residence period is compressed. Uneven surplus biomass dispersal could direct inadequate nutrient feeding within the filter bed, the primary concern with packed beds. Furthermore, the heterogeneous diffusion of surplus biomass also reduces microbial performance. For packed-bed reactors, optimizing the designs contains rinsing out the extra biomass, remixing the packing media, and adjusting the biofilter technique.^115^

### 6.4 Effect of Temperature

The effect of temperature on the performance of the biofilter was studied by heating the inlet air stream. Since the biofilter was operated for about 7–9 h daily, it never achieved a uniform temperature. Therefore, the temperature was studied by considering each bed section separately. The inlet air stream was heated to 31.5, 49, 58, and 65 °C. At each inlet temperature, the average temperature of each section in the bed and the inlet and outlet concentrations of each section were measured. Then, the elimination capacity of each bed section was determined as related to the average temperature. This indicates that the resident microorganisms were mesophilic, which grow best at a temperature range of 25–40 °C with maximum activity at 37 °C.

A review of toluene removal rates at various working temperatures exhibited maximum toluene dilapidation rates somewhere between 30 and 35 °C. Likewise, this is suggested as the ideal temperature for the expulsion of BTEX.^116^

### 6.5 pH

pH similarly affects the biofiltration compared to temperature. In an ideal pH array, bacterial action is seriously impacted in biofiltration as the more significant part of the organisms in biofilters are neutrophilic. The results of bacterial dilapidation in a biofilter are, for the most part, organic acids (e.g., acidic corrosive). Oxidation of halogenated organics and decreased sulfur amalgams (such as H_2_S) can create inorganic acid derivatives. Additionally, pollution with heteroatoms is likewise changed over acid products, reducing pH. The buildup of these acids can diminish the pH of the bed media under a vigorous pH range for bacterial dilapidation.^117^ A drop in pH can also led to additional CO_2_ and intermediate creation. To defeat this issue, buffering constituents like calcium carbonate, limestone, and so on are typically added into the bed (such as biofilters processing smelling salts fume). Alhough biofilters utilizing acidophilic microorganisms to degrade H_2_S might tolerate a lesser pH. A review of pH during BTEX degradation exhibited that maximum dilapidation was seen at pH somewhere between 7.5 and 8.0. However, for alkylbenzene degradation, it was somewhere in the range of 3.5–7.0.^118^

### 6.6 Effect of Shutdown Periods

Biotrickling filters for air corrosion management are anticipated to meet varying circumstances or times without contaminant collection. When the biofilter was shut down for specific periods and then restarted, the existing microorganisms required time to reach their maximum activity again. This period is called the “reacclimation period”—the effect of shutdown periods on the reacclimation periods of microorganisms.^119^ It is clear also that the reacclimation periods were dependent on the inlet concentration of benzene and the gas velocity (or EBCT). The biofilter was operated 7–9 h daily; thus, it involved a daily shutdown period of about 16 h. After this period, the microorganisms required about 0.5–1.0 h to degrade benzene at the highest biodegradation rate under the prevailing conditions. This period was observed where the EBCTs were 1.0 and 1.5 min, and the benzene concentration was less than 1.6 g/m^3.^^120^ Higher concentrations and shorter EBCTs required extended reacclimation periods to reach the maximum removal efficiency. The reacclimation period is crucial as it represents the length of the period during which the biofilter emits pollutant concentrations higher than the environmental regulations permit. Therefore, it should be as short as possible. This can be achieved by shortening shutdown periods. This problem is not found in plants operating continuously with periodic shutdowns.

### 6.7 Pressure Drops across the Bed (Cost-Determining Factor)

Pressure drop across the bed is an essential item in determining operating costs. Higher pressure drops result in more power consumption. Pressure drops were measured at various gas velocities both at the start of the operation and after four months to determine the effect of long-term operation; the pressure drop increased at high gas velocities (short EBCTs). Furthermore, at a specific gas velocity (or EBCT), the pressure drop across the bed increased after four months rather than at the start by a factor of 1.8. If the pressure drop value is 2500 Pa/m, the bed needs to be repacked or the compost replaced. Pressure drops of the compost used in this study were low compared to the activated carbon medium for toluene removal. Power requirements can be estimated using pressure drop results (power = flow rate × pressure drop). At an EBCT of 1.0 min and after four months, the pressure drop was 386 Pa/m. This value is equivalent to about 6.4 W per m^3^/min (or 0.182 W per cfm). This value is small compared to wet chemical scrubbing (1 W per cfm) and soil beds (0.6 W per cfm). This provides evidence that biofiltration has the advantage of low energy requirements. The pressure drop across the biofilter bed was small compared to conventional advanced process control (APC) methods.^52^

A considerable pressure reduction across the biofilter may result in air channeling into the bed. It will also improve the blower ability necessity. Causes of pressure drop are as follows: (1) increase in dampness, (2) pore size reduction in the bed, and (3) accumulation of biomass. According to research, evaporation and stripping in a biofilter handling high concentrations of contaminants may result in water losses of up to 70 g per day per kg filter bed.

### 6.8 Nutrient Necessity

Aerobic bacteria within biofilter media necessitate nutrients like nitrogen, phosphorus, potassium, sulfur, and minor components, such as additional oxygen and carbon for their development. However, the biofilter media have remaining nutrients; other nutrients are required for the long-term performance of biofilters.^121^ Subsequently, nitrogen is the second most significant component in the biomass after carbon; expanding nitrogen to the biofilter media may significantly broaden the biofilter’s performance. An investigation of a biofilter processing toluene showed that its performance powerfully depends upon the nitrogen source, and they proposed a stoichiometric mass proportion of 3.8, accepting that microorganisms controlled 13% of their mass as nitrogen and 50% as carbon.^122^

### 6.9 Inlet Pollutant

Metropolitan regions usually belong to IAQ; air pollution poses a problem to human health. Around seven million humans have died due to air pollution worldwide. People spend about 80%–90% of their life in indoor atmospheres. Therefore, indoor surroundings like academies, residences, and nursing homes have been studied. One of the essential segments of air pollution is VOCs; their indoor absorption is relatively better than the ambient atmosphere. VOCs are chemically multifarious and known to have from 10 to 100 distinct combinations, which may induce side effects like cancer, asthma, and allergies.^123^

Fixation biofilters perform best while treating a toxin that is less than 1000 ppm. Higher bay toxin fixations will prompt substrate hindrance, restraining the microbial action.^124^ Additionally, higher channel fixation will likewise lack oxygen accessibility. Scientists have found that 30 ppm of toluene had an evacuation proficiency of 99%. Yet, while the focus was multiplied, the effectiveness diminished to 82%. Additionally, investigations propose that at lesser contamination fixation, the disposal limit was seen to be lower when contrasted with a higher toxin focus in a discrepancy biofiltration container utilizing manure as the bed media.

### 6.10 Maintenance

Quickly enhancing automation has adversely impacted the atmosphere owing to water and air grade deterioration. The constant accumulation of dangerous compounds, vapor pollutants, and PMs in the atmosphere inflict life-threatening issues on flora and fauna. There is an acute necessity to assume sustainable technologies to decrease the contamination arising from air and water origins. Recently, biofiltration-based techniques have appeared, encouraging abatement methods to dismiss the unsafe impurities from wastewater or polluted atmosphere.^125^ A biofiltration framework is occasionally required, particularly during the commencement interaction. Also, occasional inspection of the biofilter bed for the level of dampness and supplement content is suggested.^122^ Climate can likewise influence the presentation of a biofilter. During substantial precipitation and snow, the biofilter should be observed for an overabundance of water or snow two times per day to ensure no unfriendly gas streams. Expansion of the wood bay coating upon the biofilter exterior might forestall the compaction instigated by a substantial downpour.

### 6.11 Empty Bed Residence Time

Practical and economical reduction of stinking gases from the air is essential for social and environmental problems. Biological procedures, including biofiltration, favor restorative air deodorization techniques due to high efficiency, low working prices, and subtle secondary contamination. Biotrickling filtration is a distinctive method of biofiltration, merging the characteristics of biofilters and bioscrubbers within one appliance.^126,127^ Wind stream rate and EBRT are boundaries that fundamentally affect biodegradation execution. Expanding the EBRT will deliver higher expulsion efficiencies. To further develop biofiltration execution, EBRT ought to consistently be more prominent than the time required for dispersion processes if there should arise an occurrence of low working stream rates. The vast majority of the exploration reports propose that more drawn out EBRT improves VOC expulsion efficiencies. In any case, to achieve longer EBRT, larger channel bed volumes are required. EBRT additionally relies on other working boundaries like poison fixation, biodegradability level, and accessible bed volumes.

### 6.12 Microorganisms and Acclimation Time

Bed media utilized in the vast majority of the biofilters are normal constituents such as soil, compost, and manure. They are the significant cause of bacterial growth. If an idle packing substance is utilized in a biofilter, then it requires a bacterial acquaintance before a biofilm grows, as microbes are contemplated as the substances toward contaminant dilapidation within biofilters. The selection of microorganisms is generally made according to the configuration of the contaminant.^128^ A solitary microorganism is sufficient to reduce specific contaminants. In a particular gathering of impurities, even an association of bacteria is utilized. An acclimatization time needed through the microbe for taking care of another substrate climate can require a couple of days to half a month, in general.^129^ The degrading classes in biofilters are typically between 1% and 15% of the all-out bacterial growth. A significant part of the biofiltration investigation has been focused on microorganisms, although fungi have also been studied. Manure has been described to utilize microbes such as *Proteobacteria*, *Actinobacteria*, *Bacteroidetes*, and *Firmicutes*. Although controlled data are accessible on the bacterial networks associated with biofiltration, novel machinery, for example, denaturing gradient gel electrophoresis (DGGE), temperature gradient gel electrophoresis (TGGE), and single-strand conformation polymorphism (SSCP), have permitted for a superior consideration of bacterial growth dynamics within open and closed biofilter arrangements.

### 6.13 Shredding/Sloughing

When a specific layer or portion of a microbe does not get sufficient nutrients and water supply, they die, and that weaker section shreds off from biomass media and comes out with the effluent; thus, shredding is good for biofilters as it keeps the media open and clean and also inhibits ponding.^130^

#### 6.13.1 Factors That Affect the Rate of Shredding

The factors that affect the rate of shredding are as follows:

- Organic loading rate (OLR): An increase in organic matter loading rate will increase microbial growth rate, resulting in the thickness of biomass portion; hence, shredding frequency increases.
- Hydraulic loading rate (HLR): Shredding frequency can also result from increased water loading pressure, resulting in prior without proper biomass growth.
- Oxygen diffusibility: More penetration of oxygen deep inside the biomass gives aerobic conditions to microbes and thus the rate of shredding frequency.
- Temperature: Microbial activity increases with increased temperature, increasing biomass thickness rapidly, thus increasing shredding frequency.

### 6.14 Role of Rodents

A decent rodent monitor program is fundamental to secure biofilters. Luckily, most cattle and poultry tasks have excellent rodent controller programs that may be passable about biofilters. Mice and rodents tunnel in cold weather via warm media, instigating channelling and poor air percolation. Rabbits, groundhogs, and badgers have been associated with tunnelling and cuddling in biofilters. Joining a biofilter to an existing rat control program is essential and low cost.^131^

## 7 Advantages and Disadvantages of Biofiltration Technique

**Figure 8:**
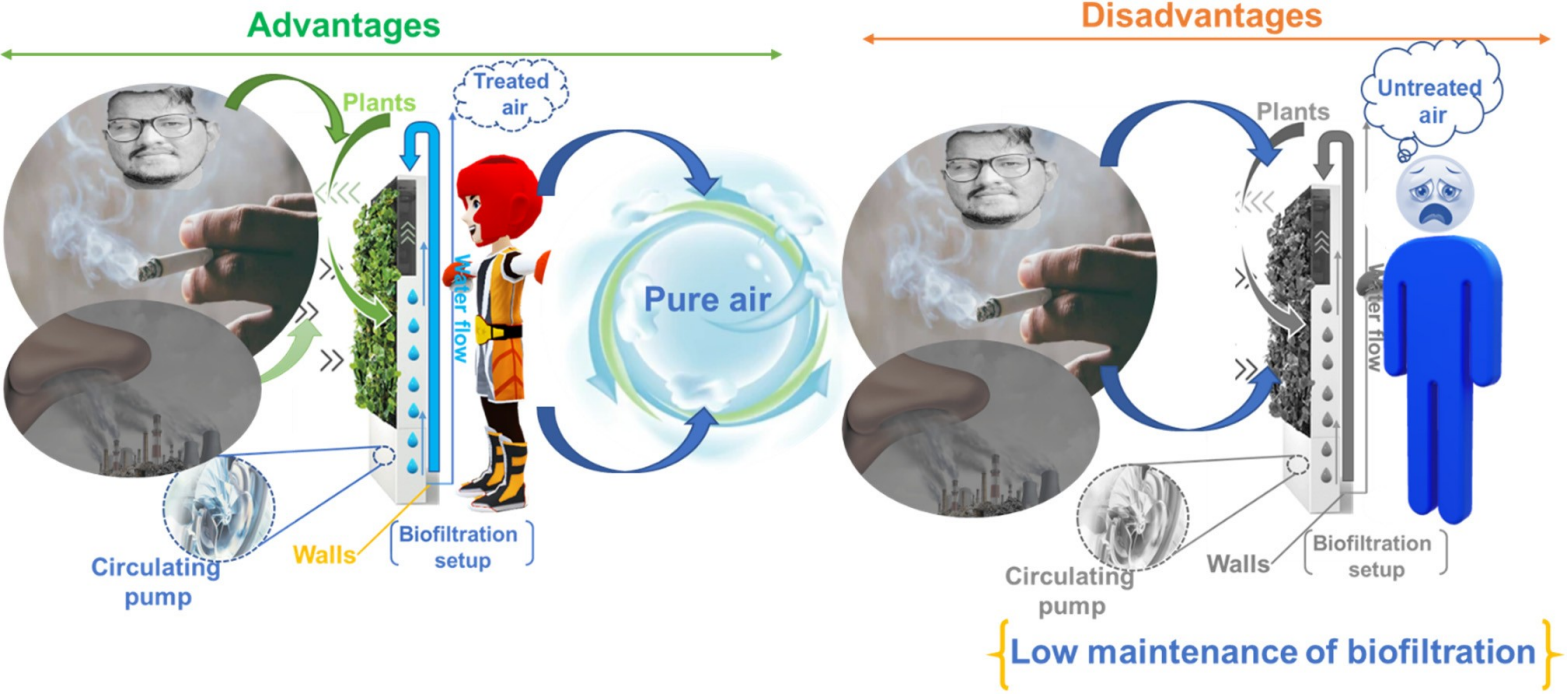
Advantages and disadvantages of biofiltration techniques.

The advantages of the biofiltration technique are low operational expenditure, lower care, and compared to wet scrubbing the filter does not deliver a contaminated water rivulet. Nevertheless, biofiltration has some disadvantages, such as essential complicated water and air diffusion approaches, backwash conditions, infrequent huge biofilm sloughing, and an elevated nitrite residue within the effluent. Figure 8 shows the advantages and disadvantages of the biofiltration techniques used for air pollutant removal.

### 7.1 Advantages

It is cost effective as less cost is required in construction and management. Also, low energy is needed and this is beneficial to the environment. One of the significant benefits of utilizing a biofilter is that it can deal with advanced inlet gas flow rates of 100–100,000 m^3^ h^–1^ compared to other air contamination regulator machinery. However, while the flow rates are too high, the residence time becomes more limited, leading to incomplete biodegradation.

A significant benefit of biofiltration is that the feasibility of microbes is kept up with for a more drawn out period. However, the framework is not in work for a more extended period.^132^ This is a result of utilizing natural constituents as the filter bed. The dependability of biofiltration for the processing of VOCs has been confirmed in a massive number of articles as it is more appropriate to process a low absorption and high volume of VOCs in a profitable method. Additionally, biofilters are great at caring for poorly soluble pollutants in water because of the better superficial area accessible for mass transfer.^133^

### 7.2 Disadvantages

It reduces its activity when not in use; i.e., in the shutdown period and when loading of gas is for a short period, they survive by endogenous respiration as they do not get nutrients from the environment. Filter beds require glucose to attain a high removal rate after shutdown. The capacity of a slip feed system to keep up with the impurity degradation movement of the biomass in a vapor phase bioreactor during starvation or shutdown periods was observed, and the system could significantly reduce the reacclimation time needed by the reactor following a shutdown period.^134^

A biofilter is not well suited for sudden changes as industrial operations have variable changes in which products changing daily or weekly are not suitable for biofilters. Also, it needs pilot plants to determine the retention time of contaminants for effectible removal. Organic packing material can degrade more in comparison to VOCs by microbes with compaction of packing material, thus increasing the pressure drop of contaminated gas. With VOC elimination limits of more than 100 g/m^3^ h, it might be hard to keep an appropriate moisture level in an extensive system, even with automatic measurement and controls.^135^

Selection of products should be made carefully for degradation as many products partially decompose and convert into more harmful byproducts. The aerobic dilapidation of trichloroethylene may form vinyl chloride as a side effect. Ductwork potential corrosion is because of moisture in the gas stream.^136^ One of the most well-known functional issues in conventional biofilters processing of CS_2_-contaminated vaporous rivulets is the lethargic beginning phase of the procedure. This is because of the bacterial poisonousness of CS_2_ and the fact that the biodiversity of microorganisms proficient in metabolizing CS_2_ seems very limited.

If the flow rate is higher, the water within the biofilter bed will be taken away by the flow, causing the biofilter to dry out: (1) Traditional biofilters have a low degradation rate. (2) The microbial community may require weeks or even months to acclimate, especially in the case of VOC treatment.^137^

Operational trouble of a trickling biofilter:

- Ponding trouble: This occurs due to excess microbes present in pores and can be prevented by adding CuSO_4_, Cl_2,_ and lime.
- Odor trouble: Foul gases are prevented by adding chlorine gas.
- Fly nuisance: This is prevented by adding DDT (dichlorodiphenyltrichloroethane).

## 8 Improving Efficiency of Biofiltration

To treat higher concentrations of gases, biofilters can use carbon adsorption technique/condensation. Efficiency can be improved by adding inert packing solids to organic packing material or switching organic with inert packing material. It requires less maintenance than organic material, and the compaction problem will be solved. It will uniformly distribute gas, but it is expensive. Adding substances, for example, lime, can be used to give a buffering ability to the bed, particularly assuming that the bed is utilized to process chloride or sulfide compounds that may bring about acidic disintegration items. Activated carbon may likewise be added to develop the contaminations further and keep a reliable feed for the microbes in cases where the interaction does not release a consistent degree of contaminants.^138^

The concentrations of VOCs are significantly less in air pollutants; therefore, the biofiltration rate depends on VOCs concentrations and is a first-order reaction. On shifting the reaction from first order to zero order, the concentrations of VOCs can be increased. This will provide more nutrients to the microbes and, consequently, a more efficient filtration process.

This natural model expects no communication between numerous contaminations in the gas stage. Since media substitution is unavoidable, the framework should be planned and developed with sufficient room and access for the vast hardware expected to “cushion” the biofilter substance or supplant it. Investigations have revealed that intermittent backflushing of the channel with water might be valuable in lessening the measure of abundant biomass that develops in the channel after some time, expanding the tension drop.^139^Table 1 demonstrates the types of biofilters and treated pollutants with their removal efficiency.

**Table 1:** Types of Biofilter-Treated Pollutants with Their Removal Efficiencies

Type of filter	Pollutant treated	Reported removal efficiency (%)	Inlet concentration (ppm)	Size of filter	ref
Full-scale packed-bed biotrickling filter	NH_3_	82	14	–	(140)
Botanical biofilter	PM	PM_10_ = 53.51	–	0.25 m^2^	(141)
		PM_2.5_ = 48.21			
Biofilter	H_2_S	79–89	38.7–48	–	(142)
	NH_3_	57–80	5.3–8		
Botanical biofilter	PM	PM_0.3–0.5_= 45	19.86	0.25 m^2^	(143)
		PM_5–10_ = 92.46	8.09 μm^–3^		
Botanical biofilter	Methyl-ethyl-ketone	56.60	30 ppbv	30 m^3^	(144)
Botanical biofilter	PM	PM_2.5_ = 54.5 ± 6.04	–	–	(12)
		PM_10_ = 65.42 ± 9.27			
	VOC	VOC = 46 ± 4.02			
Stump wood chips–bark–compost bed based biofilter	VOC	VOC= 97%	–	–	(13)
	NH_3_	NH_3_ = 99%			
	H_2_S	H_2_S = 99%			
Botanical biofilter	NO_2_	NO_2_ = 71.5%	–	0.25 m^2^	(145)
	O_3_	O_3_ = 28.1%			
	PM_2.5_	PM_2.5_ = 22.1%			

## 9 Future prospects

Biological machinery for reducing contaminants within air rivulets offers more financial benefits than physicochemical techniques, as indicated through the industrial usage of bacterial biofiltration in the previous years. Therefore, while the organic contaminants to be feted are hydrophobic, the activities of bacterial biofilters in terms of removal capability and inlet limitation are generally lower than achieved within fungal biofilters. Established biofiltration effectively removes particular contaminants from function gases as per other publications.^23^ Different outcomes, such as the biotreatment of ammonia, may be complicated. At the same time, input air has not been preprocessed, as high ammonia doping rates are related to bacterial inhibition directing to a fall in treatment implementation. Attention to free ammonia into the substrate material may hinder physical performance. The reduction capability of standard biofilters is not very effective compared to the biofiltration techniques.

Additionally, even sensible ammonia absorptions can impede the reduction of odorous VOCs. It should also be considered that there were ammonia and hydrogen sulfide within the completed experiment. Likewise, H_2_S may induce adverse consequences upon biofiltration of other contaminants due to its substrates’ inhibitory effects, which collect into the bed. Different states of urban greening are related to various outcomes upon atmospheric air corrosion concentrations. Acquiescent green fences have been suggested as an appropriate green infrastructure for lessening PM concentrations via PM deposits on plant foliage without impacting the air interaction between the street and air beyond it.

Similarly, thick walls can alter air pollutant flow and dispersal patterns to reduce pedestrian contaminant orientation into open-road essentials. The air quality lessening is noticed in the investigation due to biofiltration. With the help of altered and greater active biofilters, future work is required to confine the impact of these integrated devices upon ambient contaminant concentrations. While air pollution behavior within the environment is generally modeled, the idea of modeling the dispersal and behavior of “pure air” is a unique vision. Hence, investigation is required to evaluate biofilter impacts on ambient air quality honestly.

Economically rational biofilters with adequate technical innovation at a low acquisition and managing overhead hurdles are needed. This is feasible with the new appliances. Artificial intelligence (AI) has helped with this in extensive regions, including water processing. This would anticipate the activity of different adsorbents involving various kinds and amounts of pollutants within the wastewater. Moreover, coexisting reduction of contaminants in the absence of secondary contaminants and fouling development with valuable products are desired. Recent studies demonstrate^24^ that it is feasible to accomplish such a needed biological-based filtration through hybridization methods to extract contaminants from wastewater. Therefore, it is achievable to complete the most acceptable water processing biobased process managed by AI in the future.

## 10 Conclusion

In summary, despite numerous investigations on the performance of preserved plants, there is a determinate investigation on the calculation of essential characteristics of active biofilters to dismiss VOCs. The analysis documented here estimates the functioning of a biofilter concerning different air pollutant reduction efficiencies. The consequences of the proposed study significantly contribute to the quest for better practical strategies for the biofiltration techniques to purify the other gases. As per the publications, conventional biofiltration effectively removes respective contaminants from function gases. The range and approval of biofiltration have been observed from biotechnology advancements that deliver in-depth understanding concerning the design. It may optimize the procedure exclusively to accomplish high subtraction proficiencies with low energy consumption and significantly acquire these removal efficacies over long periods with little care.

## Abbreviations

H_2_S: Hydrogen sulfide
CS_2_: Carbon disulfide
VICs: Volatile inorganic compound
M/C: Moisture content
BaP: Benzo[2]pyrene
SEM: Scanning electron microscope
PM: Particulate matter
NO: Nitric oxide
CO: Carbon monoxide
O_3_: Ozone
PAN: Peroxyacetyl nitrate
NOx: (NOx = NO + NO_2_) Nitrogen oxide
RM: Relative humidity
PBLH: Planetary boundary layer height
PAH_3_: Polycyclic aromatic hydrocarbons
PCBr: Polychlorinated biphenyls
HAP: Household air pollution
IAP: Indoor air pollution
WHO: World Health Organization
IARC: International Agency for Research on Cancer
NAAQS: National Ambient Air Quality Standards
LPG: Liquid petroleum gas
POPs: Persistent organic pollutants
SO_2_: Sulfur dioxide
NO_*x*_: Nitrogen oxides
CO: Carbon monoxide
VOC: Volatile organic compound
BTEX: Benzene toluene ethylbenzene and xylene
RE: Removal efficiency
APCT: Air pollution control technologies
TBF: Thermophilic biofilter
o-DCB: 1,2-Dichlorobenzene
EBZ: Ethylbenzene
CH_4_: Methane
MBSPs: Membrane-based separation procedures
FO: Forward osmosis
ECO: Electrochemical oxidation
IAQ: Indoor air quality
GAC: Granular activated carbon
B: Benzene
T: Toluene
S: Styrene
X: Xylene
M: Methanol
C5: *n*-Pentane
C6: *n*-Hexane
C7: *n*-Heptane
EtOH: Ethanol
F: Formaldehyde
MPK: Methyl-propyl-ketone
EC: Elimination capacity
EBCT: Empty bed contact time
BTF: Biotrickling filtration
DGGE: Denaturing gradient gel electrophoresis
TGGE: Temperature gradient gel electrophoresis
SSCP: Single strand confirmation polymorphism
OLR: Organic loading rate
HLR: Hydraulic loading rate
DDT: Dichlorodiphenyltrichloroethane
